# Vestibular Dysfunction in Wernicke’s Encephalopathy: Predominant Impairment of the Horizontal Semicircular Canals

**DOI:** 10.3389/fneur.2018.00141

**Published:** 2018-03-12

**Authors:** Seung-Han Lee, Sang-Hoon Kim, Ji-Min Kim, Alexander Andrea Tarnutzer

**Affiliations:** ^1^Department of Neurology, Chonnam National University Hospital, Gwangju, South Korea; ^2^Department of Neurology, Chonnam National University Medical School, Gwangju, South Korea; ^3^Department of Neurology, University Hospital Zurich, Zurich, Switzerland; ^4^University of Zurich, Zurich, Switzerland

**Keywords:** vestibulo-ocular reflex, Wernicke encephalopathy, thiamine deficiency, head-impulse test, bilateral vestibulopathy

## Abstract

**Background:**

Wernicke’s encephalopathy (WE), a metabolic disorder due to thiamine deficiency, manifests with various neurological symptoms and signs. It has been known as a cause of vestibular dysfunction. Preliminary reports have proposed predominant involvement of the horizontal semicircular canals (HSCs).

**Objective:**

To better characterize the pattern of vestibular impairment in patients with WE using quantitative video head-impulse testing and to review the literature regarding this topic.

**Method:**

From January 2014 to December 2016, we retrospectively enrolled five cases of WE that received quantitative video-head-impulse testing (vHIT). We retrieved the clinical features from the medical records and reviewed quantitative head-impulse testing (qHIT) and caloric irrigation. Based on the gain and the number of corrective saccades, the function (normal vs. impaired) of each semicircular canal was rated. In addition, we conducted a MEDLINE and EMBASE search to identify other published cases of WE that had received qHIT. Neuro-otologic and neuro-ophthalmologic findings and vestibular testing results were extracted.

**Results:**

A total of 17 patients (own series = 5; published cases = 12) aged 54.6 ± 11 years were included. Key neurologic findings were ataxia of stance and gait (13/13, 100%), spontaneous nystagmus (7/14, 50%), gaze-evoked nystagmus (GEN) (17/17, 100%), positive bedside head-impulse testing for the horizontal canals (16/17, 94%), and memory impairment and mental changes (6/11, 54.5%). Regarding vestibular testing, qHIT (either video based or search-coil based) documented selective bilateral horizontal canal dysfunction with normal or minimal vertical canal impairment (14/14, 100%). On caloric irrigation, bilateral horizontal canal paresis was noted in most cases (10/11, 91%).

**Conclusion:**

In WE, signs of both peripheral and central vestibular dysfunction (i.e., GEN, ataxia of stance and gait, abnormal head-impulse testing) were common. Selective or predominant impairment of the HSCs seems to be the most common finding of WE likely related to enhanced vulnerability of the medial vestibular nuclei neurons to thiamine deficiency. Quantitative vHIT of all six semicircular canals is therefore a useful tool for the diagnosis and should be applied in all patients with suspected WE.

## Introduction

Wernicke’s encephalopathy (WE) was first described in 1881, and later the disease was named after a German doctor, Carl Wernicke (1, 2). At that time, the cause of WE was unknown, and it took another 50 years to link thiamine deficiency with the disease (3). Various medical conditions associated with nutritional deprivation such as hyperemesis gravidarum, intestinal obstruction, malignancy, and alcoholism may result in WE (4). WE is a potentially fatal disease that is still underdiagnosed in both adults and children. In adults, prevalence of WE lesions (0.8–2.8%) were higher than expected by clinical studies (0.04–0.13%) (5, 6). WE is more common in males (male-to-female ratio: 1.7 to 1) and the estimated mortality is 17% (2, 5). These numbers emphasize the need for improved diagnostic testing.

The classic symptom triad of WE consists of mental status changes, ophthalmoplegia, and gait ataxia (2, 4). However, the complete triad may be present in as few as 16–19% of cases (6, 7). For diagnostic purposes, therefore, requiring all three findings will result in low sensitivity. This is taken into account by published diagnostic guidelines such as the EFNS guidelines (8), requiring only two out of four signs (dietary deficiencies, eye signs, cerebellar dysfunction, and either an altered mental state or mild memory impairment).

Both horizontal, vertical, and gaze-evoked nystagmus (GEN) (unilateral or bilateral) abducens palsy (eventually progressing to complete external ophthalmoplegia) and internuclear ophthalmoplegia may be found (9). In a large case series with 232 WE patients, nystagmus was the most commonly (85%) described neuro-ophthalmologic finding, whereas other findings including ophthalmoplegia were less often reported (5). For detecting vestibular impairment, the angular vestibulo-ocular reflex (aVOR) can be assessed. This can be achieved by the horizontal head-impulse test at the bedside (10) or by quantitative aVOR measurements. Bilateral and often severe impairment of the horizontal aVOR is characteristic of WE and has been quantified using caloric irrigation and rotational chair testing in the past (11, 12). However, these studies were limited in the assessment of peripheral-vestibular function, as no testing of the vertical canals was possible. With the recently developed video-head-impulse testing (vHIT), quantitative assessment of all six semicircular canals became available to the clinician (13, 14). Its reliability and value in the emergency setting has been demonstrated before (15). As WE is a potentially reversible vestibulopathy if thiamine replacement is initiated in a proper and rapid manner, using the vHIT on the ED may provide very useful in assessing any vestibular impairment (16).

These considerations have fueled research interest in vestibular dysfunctions of WE, potentially supporting the diagnosis by providing a specific pattern of semicircular canal impairment. However, so far only few studies with small sample sizes have been published on this topic. Based on preliminary data from small case series and single case studies, relative sparing of the vertical canals seems to be a typical feature of vestibular impairment in WE. To increase the number of published cases and to further advance on this topic, we screened our own vHIT-database for WE patients and assessed the pattern of semicircular canal impairment. In addition, we will review and summarize the literature regarding this topic.

## Materials and Methods

### Patient Selection

We searched the electrical medical recording system for patients presenting to the emergency department or the outpatient clinic of the Department of Neurology, Chonnam National University Hospital, Gwangju, South Korea that received a diagnosis of WE. Between January 2014 and December 2016, we retrospectively identified 11 WE patients who received quantitative vestibular testing. Five consecutive patients were eligible. Six patients had to be excluded because of incomplete data (missing vHIT, *n* = 5) or completely resolved symptoms and signs at the time of testing (*n* = 1). Eventually, five patients with WE with typical history (i.e., chronic alcohol abuse, poor feeding due to gastrointestinal surgery) and laboratory (i.e., low thiamine levels), neurologic (i.e., spontaneous or GEN or ophthalmoplegia, ataxia, impaired memory or mental change), and radiologic [brainstem, mammillary body and/or thalamic lesions on T2, fluid-attenuated inversion recovery (FLAIR) or diffusion-weighted imaging (DWI) on magnetic resonance image (MRI)] findings were enrolled. As this was a retrospective case series, no pre-defined diagnostic criteria for inclusion were available and we had to rely on the treating physicians’ final diagnosis.

Video-oculography (VOG; SLMed, Seoul, South Korea) was performed in a sitting position for the detection of spontaneous (horizontal or vertical) nystagmus and GEN. All subjects received a detailed neurologic examination and vHIT and did not show evidence of central or peripheral vestibulopathy. This study was carried out in accordance with the recommendations of the Institutional Review Board of the Chonnam National University Hospital (Gwangju, South Korea) with written informed consent from all subjects. All subjects gave written informed consent in accordance with the Declaration of Helsinki. The protocol was approved by the Institutional Review Board of the Chonnam National University Hospital (Gwangju, South Korea).

### Caloric Testing

The caloric stimuli comprised alternating irrigation for 60 s with cold and hot air (24 and 50°C; 8 L/min). Nystagmus was recorded binocularly using VOG. Bilateral vestibulopathy was defined as an overall absolute slow-phase velocity of the nystagmus of less than 20°/s for all four stimulation conditions together (17).

### Video-Head-Impulse Testing

A structured bedside neuro-otologic examination was obtained from all patients at the emergency department or in the dizziness clinic of the Chonnam National University Hospital. Quantitative vHIT was obtained by use of a lightweight, portable VOG device (ICS Impulse; Otometrics, Taastrup, Denmark). Therefore, patients were asked to look at a distant target (~1.5 m away) while seated. For calibration of eye position, laser targets projected from the goggles were used. Afterward, the examiner applied a series of horizontal head-impulses to the left and right in random order. Vertical head-impulses were applied along the left-anterior–right-posterior canal plane and along the right-anterior–left-posterior canal plane (13). We aimed for head velocities between 150 and 200°/s and head displacements of 10–20°. For each canal, 20 valid head-impulses were required. Gains of the vHIT recordings were analyzed using OtosuiteV 4.0 (Otometrics). The VOR gain was calculated as the ratio of cumulative slow-phase eye velocity over cumulative head velocity from the onset of the head impulse to the moment when head velocity returned to 0 (13). This software visualizes all compensatory saccades to ensure accurate characterization. Overt saccades were defined as saccades that occurred in the opposite direction of the head rotation and that reached peak acceleration after the head had stopped moving. Covert saccades, on the other hand, reached peak acceleration before the head had stopped moving (18). Traces with artifacts (e.g., blinks during the head-impulse) were removed interactively (19). If spontaneous nystagmus (SN) was present, OtosuiteV 4.0 was operated in the nystagmus-adjusted interpretation mode. Thereby filtering algorithms for determining inadequate impulses are adjusted and traces with SN otherwise removed because of high velocity saccades will be considered as well. This makes the algorithm more robust in the presence of SN, but at the same time bears the risk of slightly less accurate aVOR gain calculations if saccades occur during the aVOR. Noteworthy, a distinction from early (covert) catch-up saccades (CS) is usually readily possible. Therefore, visual inspection as done for all traces as part of the overall rating of vHIT will ensure inappropriate traces may still be removed. All vHIT traces were independently reviewed by two experienced neuro-otologists (Seung-Han Lee and Alexander Andrea Tarnutzer). Reviewers were blinded to the clinical findings and the results from MR imaging. We used the cutoff values in VOR gains as proposed by the manufacturer of the video-goggles (Otometrics), i.e., 0.8 for the horizontal canals and 0.7 for the vertical canals. These values were also in agreement with normative values for a wide range of ages reported (20). The video-head impulse traces were evaluated by the reviewers for reduced VOR gain, increased corrective saccades, or a combination of both (21) and rated as either normal or impaired.

### Neuroimaging

According to the imaging protocol of the Chonnam National University Hospital, all patients suspected to have WE underwent MR imaging at the emergency department or the dizziness clinic of the Department of Neurology. The MRI protocol consisted of axial DWI, axial FLAIR, axial gradient-echo sequences, and time-of-flight MR angiography in a sequential manner. MR-sequences were analyzed independently by two neurologists (Seung-Han Lee and Sang-Hoon Kim), who were blinded to the clinical data. Discrepancies were resolved by consensus.

### Review of the Literature

We conducted a review of the literature on WE. We searched MEDLINE (*via* PubMed) and EMBASE using the following terms: WE, thiamine deficiency, Wernicke–Korsakoff syndrome, vestibular, dizziness, vertigo, ataxia, and nystagmus. This literature search was conducted in November 2017. Articles were selected using predetermined criteria. These criteria excluded reports that were not written in English language, did not include human subjects, lacked original patient data, did not provide a description of vestibular symptoms, or did not indicate vestibular function testing. For inclusion, thiamin-deficiency and confirmed bilateral vestibulopathy (either by caloric irrigation or head-impulse-testing) were required. From suitable cases, we extracted both clinical data and results from vestibular testing [caloric irrigation and/or quantitative head-impulse testing (qHIT)].

We identified 13 articles (out of 1,167), 8 of which were excluded due to either the presence of only descriptive vestibular symptoms and/or tests (i.e., caloric irrigation) without reporting head-impulse data (*n* = 7) or duplicated data (*n* = 1). After a full-text review, we found 5 manuscripts reporting on a total of 12 cases with WE that included head-impulse testing of both the horizontal and the vertical canals (16, 22–25). From one publication, two cases were excluded due to the lack of head-impulse testing of the vertical canals (16), whereas from another publication, one case was excluded due to duplicity (24).

## Results

### Representative Case

In June 2011, a patient in the early 60s (patient #2) was referred to the dizziness clinic of the Department of Neurology, Chonnam National University Hospital, due to gait disturbance and oscillopsia. In May 2010, he had a laparoscopic colectomy due to suspected colon cancer. About 1 month after the colectomy, an entero-cutaneous fistula developed as a complication. For about 50 days, total parenteral nutrition (TPN) and antibiotic treatment were performed. Fistulectomy and extended right hemicolectomy were performed in August 2010 and TPN was stopped again. In January 2011, the caregivers noted an impairment of memory. However, at that time no further evaluations were performed. When referred to the dizziness clinic 5 months later, the initial neurologic examination revealed a recent memory impairment with normal mental status, GEN and gait ataxia. Also, the bedside head-impulse test for the horizontal canals showed bilateral CS, and bithermal caloric irrigation demonstrated bilateral canal paresis (see Tables 1 and 2). Brain MRI demonstrated atrophy of the mammillary bodies, with increased signal intensity on FLAIR imaging, suggestive of chronic WE. From the time, the diagnosis of WE was established, one of the authors (Seung-Han Lee) followed-up the patient and regularly assessed vestibular function. Whereas the patient received ambulatory vestibular rehabilitation, the bedside head-impulse test remained abnormal bilaterally. In October 2014, vHIT was performed, demonstrating low gains and clear CS (overt and covert) in both horizontal canals. In February 2016, the vHIT was repeated, indicating persistent impairment of the horizontal canals.

**Table 1:** Demographical and clinical findings of five patients with WE.

#	Age range (years)	Cause of WE	D/V	M/M	Op	G/S	SN	GEN	bHIT for HC	Thiamine (initial)^b^	Abnormalities on brain MRI	Thiamine replacement	Recovery
1	66–70	Alcohol	Y	Y^a^	Partial bilateral 6th palsy	Y	UB	Y	CS/CS	33.78	T2/FLAIR lesions in MVN, PAG, MB, HT, medial thalamus	IV (1,500 mg/day for 3 days, then 250 mg/day for 4 days) followed by PO (thiamine HCL 30 mg/day for 22 months)	All symptoms resolved after 6 months (22 months F/U in total)
2	66–70	Gut OP/TPN	Y	Y	N	Y	N	Y	CS/CS	NA	FLAIR lesions in MB and atrophy of MB	PO (thiamine HCL 20 mg/day + benfotiamine 138.3 mg/day for ~5 years)	Not improved (~6 years F/U in total)
3	36–40	Alcohol	N	N	N	Y	N	Y	CS/CS	NA	FLAIR lesions in MB and PAG	IV (1,500 mg/day for 7 days), followed by PO (thiamine HCL 30 mg + benfotiamine 138.3 mg/day for 21 days), then (thiamine HCL 20 mg/day + fursultiamine 54.57 mg/day for 20 months)	G/S—persistent (2.5 years F/U in total); others—improved
4	66–70	Alcohol	N	Y	N	Y	N	Y	CS/CS	56.17	T2/FLAIR lesions in PAG, medial thalamus, MB; atrophy of MB	IV (750 mg for 7 days) → PO (thiamine HCL 90 mg + benfotiamine 138.3 mg for 3 months) → (thiamine HCL 40 mg/day + benfotiamine 138.3 mg/day for 7 months)	Mental, G/S—improved; memory, HIT—persisted during 9 months F/U
5	46–50	Alcohol	N	N	N	Y	UB	Y	CS/CS	51.23	NS	IV (600 mg/day for 10 days), followed by PO (thiamine HCL 90 mg/day + benfotiamine 138.3 mg/day for 1 month)	Complete
All	58.2 (±15)	Alcohol (*n* = 4), TPN (*n* = 1)	2/5	3/5	1/5	5/5	2/5	5/5	5/5	47 (±11.7)	4/5	IV + PO (*n* = 4), PO only (*n* = 1)	Complete (*n* = 2), partial (*n* = 2), none (*n* = 1)

*^a^Mild memory impairment*.

*^b^Normal range of total serum thiamine levels for this study was 66–200 nmol/L*.

*bHIT, bedside head-impulse testing; CS, catch-up saccades; D/V, dizziness and/or vertigo; F, female, FLAIR, fluid-attenuated inversion recovery; GEN, gaze-evoked nystagmus; G/S, gait and station impair; HT, hypothalamus; IV, intravenous; M, male, M/M, memory impairment or mental change; MB, mammillary body; MVN, medial vestibular nucleus; N, no; NA, not available; NS, non-specific; Op, ophthalmoplegia; PAG, periaqueductal gray matter; PO, per oral; SN, spontaneous nystagmus; TPN, total parenteral nutrition; UB, upbeat nystagmus; WE, Wernicke’s encephalopathy; Y, yes; MRI, magnetic resonance image*.

### Clinical and Laboratory Findings in Our Patients

A total of five patients (four men and one woman) with a mean age of 58.2 years (SD = 14.7 years) were included in this retrospective data analysis. The causes of thiamine deficiency were alcoholism (four out of five) and history of gut surgery with TPN (one out of five).

Neurologic examination showed memory impairment and/or mental changes (three out of five, 60%), gait ataxia (five out of five, 100%), partial bilateral sixth nerve palsy (one out of five, 20%), GEN (five out of five, 100%), spontaneous upbeat nystagmus (two out of five, 40%), and positive bedside head-impulse testing for the horizontal canals (five out of five, 100%). Hearing was normal in all patients and none of them reported previous audio-vestibular symptoms (dizziness, tinnitus, etc.).

**Figure 1:**
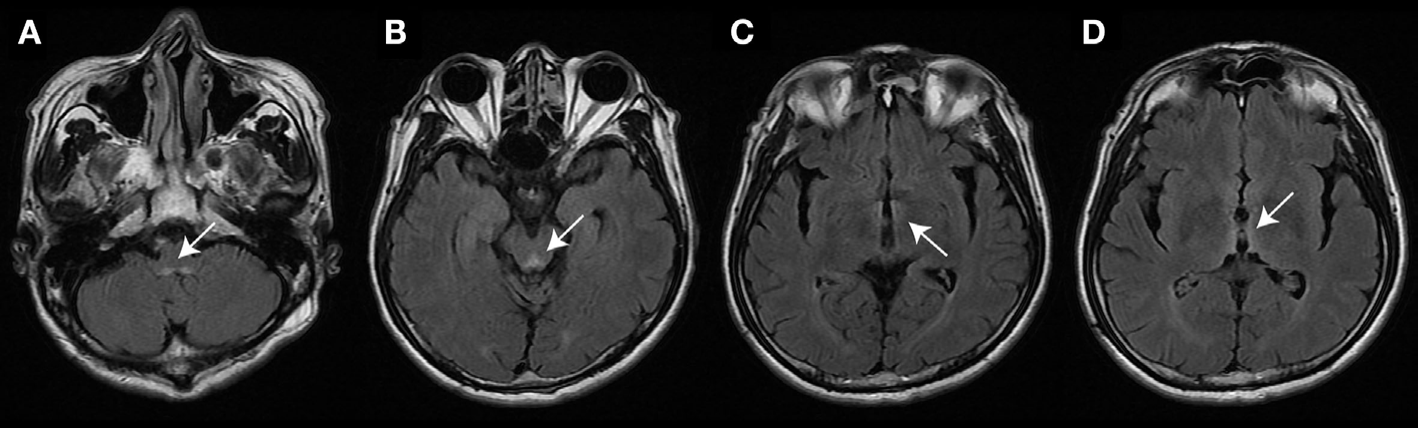
Brain magnetic resonance image (patient #1) studies (fluid-attenuated inversion recovery images) show bilateral symmetrical lesions (as marked by white arrows) in the medial vestibular nucleus **(A)**, the periaqueductal region **(B)**, around the hypothalamus and mammillary bodies **(C)**, and the periventricular regions of the thalamus **(D)**.

In three patients, the initial serum total thiamine levels were checked and found to be below normative values. Brain MRI was performed in all patients. Except patient 5, all patients showed typical imaging abnormalities linked to WE (see Table 1 for details). The MRI of patient 1 had typical lesions including the medial vestibular nucleus (MVN) (Figure 1).

### Bithermal Caloric Irrigation

Bithermal caloric irrigation was obtained in four out of five patients, whereas one patient (#5) was not tested. In three patients, the sum of nystagmus slow-phase velocities was below 20°/s, whereas in patient 3 the value was above 20°/s (Table 2).

**Table 2:** Findings of caloric irrigation and video-head-impulse testing (vHIT) in five patients with Wernicke’s encephalopathy.

#	bHIT for HC	O-to-A (d)	T-to-C (d)	T-to-vHIT (d)	SPV on caloric irrigation (°/s)				vHIT^a^ (gains and CS)								
						
					RC	RW	LC	LW	RH	LH	RA	LA	RP	LP	CS (RH/LH)^b^	CS (RA/LA)	CS (RP/LP)
1	CS/CS	8	4	4^c^	4.3	−3.0	−4.6	1.6	***0.66***	***0.67***	0.82	0.83	0.76	0.86	+/+	−/−	−/−

2	CS/CS	240	78	78	4.2	−3.6	−5.1	2.8	***0.55***	***0.68***	0.81	0.93	1.09	0.81	+/+	−/−	−/−

3	CS/CS	90	2	2	7.7	−5.4	−10	9.1	***0.49***	***0.67***	0.88	0.90	1.08	1.00	+/+	−/−	−/−

4	CS/CS	30	4	4	4.9	−2.9	−3.2	6.7	***0.77***	***0.79***	0.53	0.67	0.66	0.53	+/+	−/−	+/−

5	CS/CS	1	NA	8	NA	NA	NA	NA	0.89	***0.63***	0.73	0.71	0.93	0.81	−/+	−/−	−/−

Avg	NA	73.8 ± 99.3	22.0 ± 37.3	19.2 ± 32.9					***0.67*** ± ***0.16***	***0.69*** ± ***0.06***	0.75 ± 0.14	0.81 ± 0.11	0.90 ± 0.19	0.80 ± 0.17	NA	NA	NA

*^a^Gain values in bold and italics refer to those semicircular canals that were rated as overall abnormal by the reviewers (Seung-Han Lee and Alexander Andrea Tarnutzer)*.

*^b^CS were either present (+) or absent (−)*.

*^c^Note that at the time of vHIT the patient had recovered mostly from the bilateral sixth nerve palsy*.

*bHIT, bedside head-impulse testing; CS, catch-up saccades; d, days; LA, limb ataxia; LC, left cold; LH, left horizontal canal; LP, left posterior canal; LW, left warm; NA, not available; O-to-A, onset to admission; RC, right cold; RW, right warm; RA, right anterior canal; RH, right horizontal canal; RP, right posterior canal; SPV, slow-phase velocity; T-to-C, treatment to caloric testing; T-to-vHIT, treatment to video-head-impulse testing*.

### Quantitative vHIT

#### Analysis of VOR Gains and Compensatory Saccades

**Figure 2:**
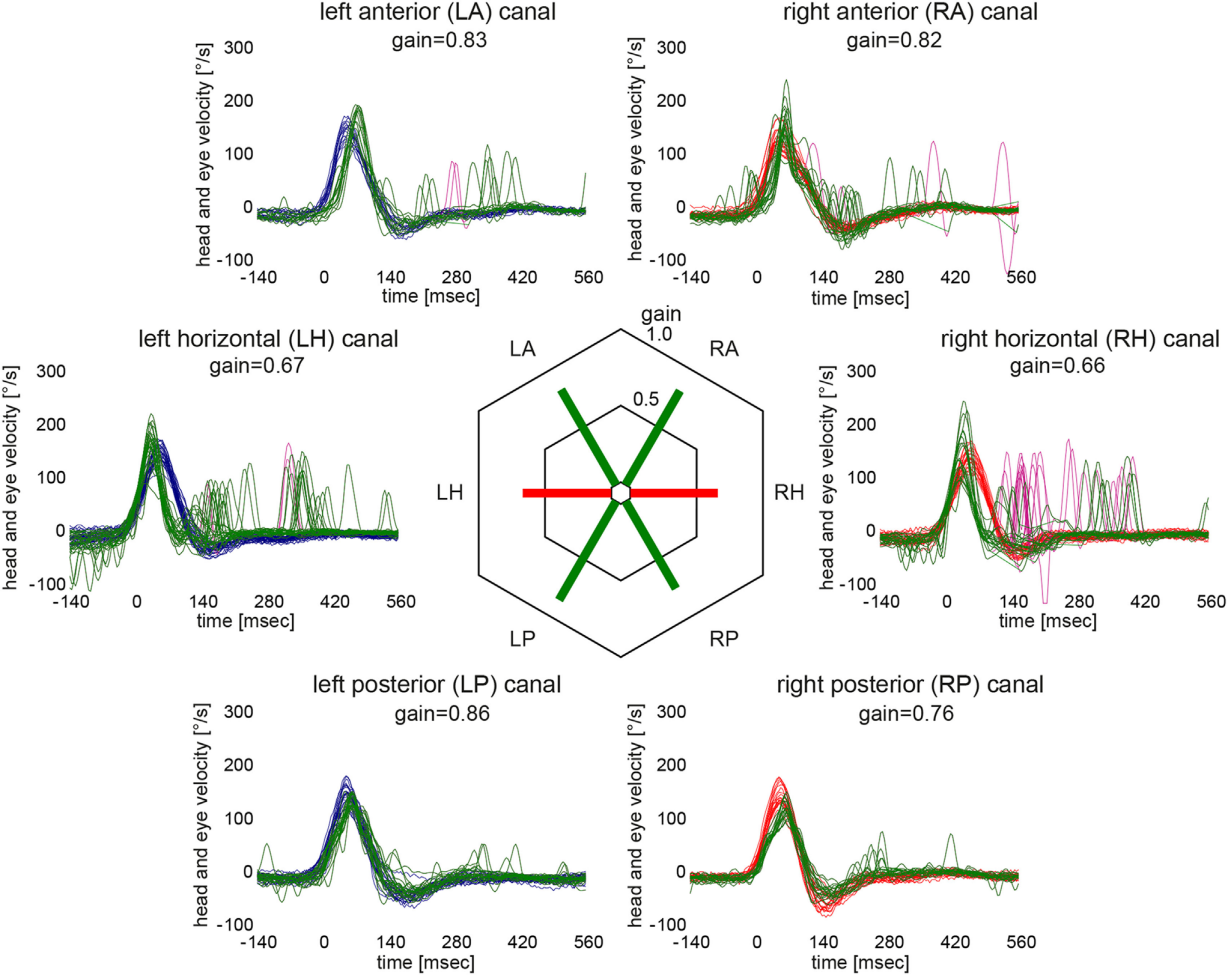
Video head-impulse testing from the same patient as in Figure 1 (patient #1) 12 days after symptom onset illustrating semicircular canal impairment restricted to the horizontal canals. For each semicircular canal, individual eye velocity traces (in green) and head velocity traces (in red for assessing the right vestibular organ and in blue for assessing the left vestibular organ) are plotted against time (20 trials per canal were recorded). Note that eye velocity traces are inverted to allow for better visualization and comparison with the head velocity traces. Mean gain values (eye velocity/head velocity) are shown in the hexagonal plot in the center of the figure. Whereas green bars indicate normal gains, red bars refer to reduced gains.

**Figure 3:**
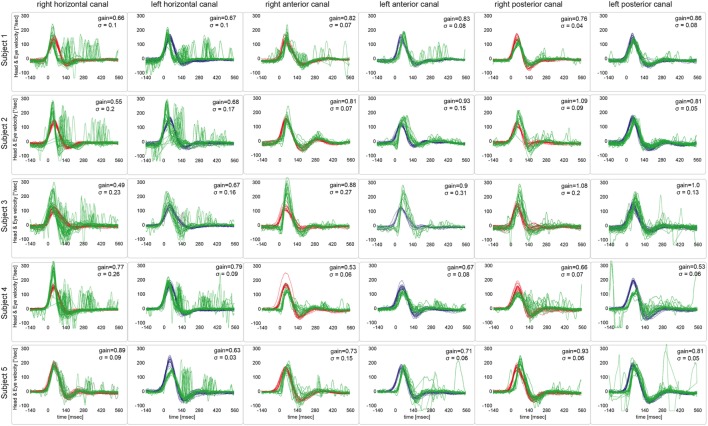
Video head-impulse testing from all five patients, showing the results from a single subject in one row. For each semicircular canal, individual eye velocity traces (in green) and head velocity traces (in red for assessing the right vestibular organ and in blue for assessing the left vestibular organ) are plotted against time. Note that eye velocity traces are inverted to allow for better visualization and comparison with the head velocity traces. While upbeat nystagmus was present at the time of vHIT recordings in patients #1 and #5, this did not result in an increased number of saccades on the traces or reduced gains.

Based on the analysis of VOR gains and compensatory saccades (Table 2), vHIT demonstrated preferential impairment of the horizontal semicircular canals (HSCs), as shown for a single patient in Figure 2. The VOR gains of HSCs were decreased (i.e., were below 0.8) in all five patients except for the right HSC in patient 5 (see Table 2 for average values and Figure 3 for individual measurements). The VOR gains of the vertical canals were mostly normal (i.e., above 0.7) except for patient 4 who showed subnormal VOR gains in the vertical canals (Table 2). Compensatory CS were observed in all HSCs except for the right HSC in patient 5. For the vertical canals, no CS were noted. This was true also for patient 4 who showed slightly reduced vertical canal VOR gains. Note that vHIT was obtained between 3 and 6 days *after* diagnosis of WE and treatment initiation with thiamine in all our patients except for patient 2 who received testing about 3 years after diagnosis.

#### Overall Function of Individual Semicircular Canals

The reviewers rated each canal function as normal or impaired without knowledge of the clinical findings and the results from MR imaging. Inter-rater agreement for individual canal function (normal vs. abnormal) in all five subjects was 0.94 (Cohen’s kappa) (26). After resolving discrepancies, both horizontal canals were rated as abnormal in four out five patients, while in one patient (#5) the right horizontal canal was spared. The vertical canals were rated as normal in all patients (see Table 1 for details).

### Literature Review

We identified five papers that reported on detailed vestibular function testing in WE patients. We excluded the duplicate data and then finally extracted data from 12 patients (53.2 ± 9.7, 6 males, 6 females) from five papers. Details are shown in Tables 3 and 4. In all 12 patients, bilateral vestibulopathy was confirmed by abnormal bithermal caloric tests (*n* = 7/7) and/or bedside horizontal head-impulse-testing (*n* = 11/12). GEN (12 of 12), difficulties in stance and/or gait (8 of 8), and diplopia (3 of 5) were common symptoms and signs of WE with bilateral vestibulopathy. qHIT (vHIT or magnetic search-coil testing) was available in 9 patients and revealed selective HSC impairments (*n* = 6) or predominant HSC impairment with minimally reduced vertical canal function (*n* = 3) (Tables 3 and 4). The timepoint of vHIT relative to treatment initiation could be retrieved only from three patients besides our five patients (see Table 4).

**Table 3:** Clinical and neuro-otologic findings in selected cases with Wernicke’s encephalopathy reporting vestibular function (data from literature review).

#	Age (years), sex	Cause	V	D	H	M/M	Op^a^	G/S	LA	SN	GEN	CPN	bHIT	Pur	Sac	CP	qHIT		B1 nmol/L	MRI	Replacement therapy	Recovery
																	Hor	Ver				
																					
**Choi et al. (22)**																						
1	63, F	TPN	Y	NA	N	Y	Y	Y	Y	NA	Y	NA	CS	Ab	Ab	Y	Ab	Ab^b^	NA	Ab	IV (100 mg/day)	Par
2	40, M	Alcohol	Y	NA	N	Y	Y	Y	Y	NA	Y	NA	CS	Ab	Ab	Y	Ab	Nr	NA	Nr	IV (dose NA)	Par

**Kattah et al. (16)**																						
3	50, F	Alcohol	N	NA	NA	N	N	Y	N	N	Y	NA	CS	NA	NA	Y	Ab	Nr	88	Nr	PO (100 mg/day)	NA
4	60, M	G.byp/Vo	Y	Y	NA	N	Y	Y	N	N	Y	NA	CS	NA	NA	NA	Ab	Nr	21	Nr	IV (500 mg/day), then PO (100 mg/day)	N
5	37, F	G.byp/Vo	N	NA	NA	N	N	Y	N	DB	Y	NA	CS	NA	NA	NA	Ab	Ab^b^	55	NS	IV (500 mg/day), then PO (100 mg/day)	Par

**Choi et al. (24)**																						
6	53, M	NA	Y	N	N	NA	NA	NA	Y	UB	Y	NA	CS	Ab	Ab	Y	NA	NA	NA	NA	NA	NA
7	64, M	NA	Y	Y	N	NA	NA	NA	N	UB	Y	Y	CS	Ab	Ab	Y	Ab	Nr	NA	Ab	NA	NA
8	45, F	NA	Y	Y	N	NA	NA	NA	N	N	Y	N	CS	NA	NA	Y	NA	NA	NA	NA	NA	NA
9	62, M	NA	Y	N	N	NA	NA	NA	N	N	Y	N	Nr	Nr	Ab	Y	NA	NA	NA	NA	NA	NA

**Akdal et al. (23)**																						
10	55, F	Alcohol	N	NA	N	NA	Y	Y	NA	UB	Y	NA	CS	Ab	Ab	NA	Ab	Nr	NA	Ab	IV (dose NA)	NA
11	64, M	TPN	Y	NA	N	NA	Y	Y	NA	NA	Y	NA	CS	NA	Ab	NA	Ab	Nr	NA	Ab	IV (600 mg for 3 days, then 200 mg for 5 days), then PO (100 mg/day)	Par

**Kattah et al. (25)**																						
12	45, F	Alcohol/Vo	NA	NA	NA	Y	NA	Y	NA	UB	Y	NA	CS	NA	NA	NA	Ab	Ab^b^	~30	Ab	IV(500 mg/day)	Par
All	53.2 (±9.7)		8/11	3/5	0/8	3/6	5/7	8/8	3/9	5/9	12/12	1/3	11/12	5/6 Ab	7/7 Ab	7/7	9/9 Ab	3/9 Ab	48.5 (±30)	5/9 Ab	7/8 IV	5/6 Par

*^a^The extent of ophthalmoparesis varied. While “limited ocular motor range” in the horizontal plane was reported in cases 1 and 2 being suggestive of partial bilateral sixth nerve palsy, case 4 had “8-prism diopter esotropia in left gaze during cross-cover testing,” “almost total loss of horizontal eye movements” in case 10 is consistent with severe bilateral sixth nerve palsy and “bilaterally limited horizontal gaze” in case 11 likely reflects partial bilateral sixth nerve palsy*.

*^b^In these patients, vertical canals were rated as borderline abnormal*.

*Ab, abnormal; ap, apogeotropic nystagmus; bHIT, bedside head-impulse testing; B1, thiamine; CP, canal paresis tested by caloric test; CPN, central positional nystagmus; CS, catch-up saccades; D, diplopia; DB, downbeat; F, female; G. byp, gastric bypass; GEN, gaze-evoked nystagmus; G/S, gait or standing impair; H, hearing loss; Hor, horizontal canal; IV, intravenous; LA, limb ataxia; M, male; M/M, memory impair or mental change; MRI, magnetic resonance image; N, no; NA, not available; Nr, normal; NS, non-specific; Op, ophthalmoplegia; Par, partial recovery; PO, per oral; Pur, pursuit; qHIT, quantitative head-impulse testing (either video based or search-coil based); Sac, saccades; SN, spontaneous nystagmus; TPN, total parenteral nutrition; UB, upbeat; Ver, vertical canal; V, vertigo; Vo, vomiting; Y, yes*.

**Table 4:** VOR gains obtained by head-impulse testing (video-oculography or search-coils) in 11 patients (5 from our series and 6 from the literature review) with Wernicke’s encephalopathy.

	Test time relative to Tx	Method	VOR gains^a^					
			
			RH	LH	RA	LA	RP	LP
**Own cases**								
# 1	Post-Tx ~72 h	vHIT	*****0.66*****	*****0.67*****	0.82	0.83	0.76	0.86
# 2	Post-Tx ~3 y	vHIT	*****0.55*****	*****0.68*****	0.81	0.93	1.09	0.81
# 3	Post-Tx ~72 h	vHIT	*****0.49*****	*****0.67*****	0.88	0.90	1.08	1.00
# 4	Post-Tx ~72 h	vHIT	*****0.77*****	*****0.79*****	0.53	0.67	0.66	0.53
# 5	Post-Tx ~144 h	vHIT	0.89	*****0.63*****	0.73	0.71	0.93	0.81

**Choi et al. (22)**								
# 1	NA	Search coils	*****0.38*****	*****0.31*****	0.84	0.66	0.72	0.8
# 2	NA	Search coils	*****0.44*****	*****0.53*****	0.76	0.7	0.85	0.86

**Choi et al. (24)**								
# 7	NA	Search coils	*****0*****	*****0.1*****	0.91	0.93	0.67	0.67

**Akdal et al. (23)**								
# 10	Post-Tx ~24 h	Search coils	*****0.18*****	*****0.13*****	0.81	0.72	0.99	1.16
# 11	Post-Tx ~4 m	vHIT	*****0.27*****	*****0.12*****	0.86	0.84	0.82	0.84

**Kattah et al. (25)**								
# 12	Pre-Tx	vHIT	*****0.43*****	*****0.26*****	*****0.51*****	0.75	0.55	0.13
# 12	Post-Tx 72 h	vHIT	*****0.60*****	*****0.51*****	0.93	0.51	0.78	0.95

*^a^Gain values in bold and italics refer to those semicircular canals that were rated as overall abnormal*.

*h, hours; LA, limb ataxia; LH, left horizontal canal; LP, left posterior canal; m, months; NA, not available; RA, right anterior canal; RH, right horizontal canal; RP, right posterior canal; Tx, treatment; vHIT, video-head-impulse testing; y, years*.

## Discussion

Contrary to common wisdom, vestibular dysfunction in WE is rather common, even though numbers on its incidence are lacking. More importantly, there seems to be a characteristic pattern of vestibular impairment in WE, with preferential loss of function of the HSCs and sparing of the vertical canals, as confirmed both in our patients and in those cases identified in the literature. With respect to previous studies reporting on the clinical findings in WE, we think that vestibular dysfunction may have been missed for several reasons. In the study of Victor and co-workers, reporting on 245 patients with WE, the authors noticed that ataxia of stance and gait was common (87%), whereas ataxia of the lower extremities was present in only 20% of the patients (5). To explain the ataxia of stance and gait, the authors therefore proposed loss of function of midline cerebellar structures including the vermis, while peripheral-vestibular function was not assessed. This has led to the perception that in WE rather co-existing cerebellar lesions caused disturbances in gait and stance than a vestibulopathy.

However, both previous studies as identified in our literature review and our own cases indicate that ataxia of gait and stance could be linked to vestibular dysfunction as well. While previous studies applied caloric irrigation or rotatory chair testing for the evaluation of vestibular function in WE (11, 12), it is head-impulse testing that has contributed to the early detection of these symptoms more recently. As vestibular disturbances can be reversible, showing rapid improvement after thiamine replacement (16, 25), this underlines the importance of testing the aVOR in these patients acutely, either at the bedside or if available quantitatively by vHIT. In case of delayed vHIT (after treatment initiation), there is a risk of missing the acute impairment of the aVOR due to recovery. Furthermore, while vertigo/dizziness and acute motion intolerance, which are common in acute unilateral vestibular loss such as vestibular neuritis or cerebellar stroke (27), are less common in WE, some WE cases presented with vertigo and/or dizziness (16, 22). This was probably due to an asymmetric or sequential impairment of vestibular function. Therefore, also in asymmetric aVOR deficits and acute vertigo/dizziness, WE should be considered.

### Vestibular Testing—Comparison of Different Tests and Limitations

Although gait disturbance and oscillopsia are frequently encountered in WE patients, deficits in the aVOR may be difficult to be detected in the acute phase since other symptoms such as ophthalmoplegia or nystagmus and limb ataxia are potential confounders, and other conditions such mental changes restrict history taking and the neurologic examination. Indeed, ocular motor palsies, either affecting a single nerve or presenting as complete external ophthalmoplegia, may limit the use of (video) head-impulse testing. While in a case series of 17 unselected WE patients (i.e., not included based on the presence of vestibular complaints such as vertigo or dizziness) vestibular impairment was reported in all patients (11), 11 patients had bilateral abducens palsy and 1 patient had total external ophthalmoplegia. In another case series, one out of two patients had bilateral sixth nerve palsy as well (12). These ocular motor deficits may have led to false abnormal caloric irrigation and rotatory chair testing results. From the 17 cases included here (own data and previously published cases), information on extraocular muscle palsies was available only in 12. Partial sixth nerve palsies were found in six patients, complete horizontal ophthalmoplegia was noted in one case. In these cases, the interpretation of vHIT and caloric irrigation must be made with caution and these tests will become useless in case of total external ophthalmoplegia as, e.g., in case 10 from Table 3. Note that in the single case from our own case series with initial partial bilateral abducens palsy ocular motor function had normalized at the time of vHIT on day four after admission.

In four out of five patients from our own data, horizontal canal impairment as assessed by caloric irrigation and by vHIT was consistent, whereas in one case (#3) a discrepancy between these two tests was noted. Such discrepancies might point to selective high-frequency aVOR impairment due to damage of the vestibular nuclei (VN) secondary to thiamine deficiency. In the paper by Choi and co-authors, one patient showed marked improvement on caloric irrigation, whereas the responses of rotatory chair testing and head-impulse testing remained unchanged (22). Similar to this report, in our patient #3, the caloric responses were near normal, whereas vHIT still showed bilateral horizontal canal impairment. This finding may be explained by the fact that caloric irrigation and vHIT were performed in the recovery period after thiamine replacement (usually within 3–6 days, but sometimes delayed by months or years). MVN neurons responsible for high acceleration horizontal aVOR may be the most vulnerable to thiamine deficiency, and this selective susceptibility may occur due to high metabolic demands of the neurons responsible for the high acceleration aVOR as proposed by Choi et al. (22).

Comparing bedside and video HIT findings in our case series, results were consistent in all but one patient (#5). While in this patient, the bedside HIT showed bilateral CS, vHIT demonstrated only unilateral CS. Noteworthy, the bedside HIT was performed at admission 1 day after symptom onset, whereas the vHIT was obtained 1 week later. Thus, in this patient the vHIT reflects partially improved vestibular function after thiamine supplementation. Obviously, prognosis depends on the delay of treatment initiation. Recovery without neurological sequelae after prompt treatment was described in four out of five patients with acute/subacute symptom onset by Kattah and colleagues (16), whereas in our case series complete recovery was noted in two out of five cases only. Noteworthy, delay from symptom onset to diagnosis was 30 days or more in three of our five patients. Permanent vestibular injury persisting more than 5 years was noted in patient 2 due to delayed diagnosis and treatment.

### MR Imaging in WE

Magnetic resonance image studies typically show bilateral symmetrical lesions in the periventricular regions of the thalamus, the hypothalamus, the mammillary bodies, the periaqueductal region, the floor of fourth ventricle, and the midline cerebellar structures (2). In our case series, abnormal MRI findings compatible with WE were found in four out of five cases (80%). However, in the pooled data analysis, only 9 of 14 WE cases who received MRI (64%) showed typical MR abnormalities. According to the literature, MRI has a moderate sensitivity (53%) only for detecting WE, while its specificity is high (93%) (28, 29). MR imaging may therefore be used to rule out WE, but a negative MRI does not exclude WE (2). Even though our series is retrospective and numbers from the literature reporting on the HIT in WE, the vHIT is likely more sensitive for the diagnosis of WE than MR imaging.

### Explanations for Vertical Semicircular Canal Sparing in WE

Consistently with the literature, we noted selective impairment of the horizontal canals. Thus, such sparing of the vertical canals in the presence of bilateral horizontal canal impairment seems to be a pattern suggestive for WE. According to previous histopathologic studies, vestibular paresis in WE may be accounted for by loss of function of the VN (12). Neuropathologic examinations of patients with WE have revealed lesions in the VN, especially in the MVN, the nucleus prepositus hypoglossi, the nodulus, and the uvula (25). The MVN was most vulnerable to thiamine deprivation (30), and histological abnormalities in the labyrinthine cristae and vestibular nerves were relatively minor in thiamine deficient pigeons (31).

The vestibular neurons receiving different primary afferent input have a topographic distribution within the VN (32). The neurons activated by the saccule, utricle, and anterior and posterior canals are located mainly in the lateral VN (LVN) and the descending VN, while the neurons activated by the lateral canal were found mainly in the MVN and the LVN. Thus, vertical canal sparing and the distinct susceptibility of the vestibular end organs in WE may result from selective vulnerability of the neurons in the MVN to thiamine deficiency (5, 22, 33). In our case series, only 4 out of 13 cases who had received structural MR imaging (one from our series and three from the literature review) showed involvement of the MVN. However, all patients had selective or predominantly horizontal canal dysfunction in head-impulse testing. Low sensitivity of MRI and functional impairment rather than structural lesion may explain this discrepancy between the results of MRI and head-impulse testing. This is also supported by the notion that supplementation of thiamine in the acute stage may result in rapid clinical improvement and normalization of the HIT (25).

### Limitations

Our paper has several limitations. This is a retrospective data analysis and therefore no prospectively defined diagnostic criteria for WE were available. Furthermore, due to relative rarity of WE, both our sample size and those from the literature were small. Also, data published were sometime incomplete (e.g., lacking raw data of search-coil or video head-impulse testing, thiamine levels or MRI). Practically, it was the combination of patient history, acute/subacute ocular motor and gait impairment, and either low thiamine levels or characteristic findings on MR imaging that led the treating physicians to a diagnosis of WE.

Importantly, ocular motor palsies as a confounder for abnormal results on HIT, caloric irrigation and rotatory chair testing were not taken into account in some cases. Vestibular dysfunction can be reversible after thiamine replacements. Therefore, timing of vestibular testing is crucial and recovery after treatment initiation may result in false negative results. Furthermore, we used an overall, reviewer-based rating of semicircular canal function as previously proposed by Tarnutzer and colleagues (21, 34), whereas in previous studies gain values were the single most important parameter for assessing vestibular function. In some of our patients, we noted artifacts on vHIT with peak eye velocities exceeding peak head velocities in predominantly the horizontal canals. This most likely reflects slippage of the vHIT-goggles and may result in false high (i.e., normal) gain values. Nonetheless, both the actual gain values in these patients and the CS clearly indicated impairment of the horizontal canals in these patients.

## Conclusion

In conclusion, bedside or video HIT is valuable tools for the diagnosis of WE especially in the emergency department because MR imaging has a relatively low sensitivity in WE and may not be readily available. In case of acute to subacute bilateral vestibulopathy, WE should be in the list of differential diagnoses. In cases with early (i.e., covert) CS, vHIT will be superior compared to bedside HIT. Furthermore, with relative sparing of vertical canal function in the presence of profound bilateral horizontal canal impairment being the most common pattern, vHIT may facilitate the diagnosis of WE and accelerate thiamine supplementation, eventually improving the clinical outcome in these patients.

## Ethics Statement

This study was carried out in accordance with the recommendations of the Institutional Review Board of the Chonnam National University Hospital (Gwangju, South Korea) with written informed consent from all subjects. All subjects gave written informed consent in accordance with the Declaration of Helsinki. The protocol was approved by the Institutional Review Board of the Chonnam National University Hospital (Gwangju, South Korea).

## Author Contributions

S-HL drafted the manuscript, performed the data collection, analyzed the data, and conceived of the study. S-HK analyzed the data and conducted the statistical analysis. J-MK performed the data collection, interpreted neuro-images, and analyzed the data. AT was involved in the design of the study, participated in the data analysis and the statistical analysis, and critically reviewed and edited the manuscript. All authors read and approved the final version of the manuscript.

## Conflict of Interest Statement

The authors declare that the research was conducted in the absence of any commercial or financial relationships that could be construed as a potential conflict of interest. The reviewer AK and handling editor declared their shared affiliation.
